# Genetic and biological characterization of a reassortant H3N2 swine influenza virus isolated in China with internal genes from the 2009 pandemic H1N1

**DOI:** 10.1186/s12866-026-05324-w

**Published:** 2026-06-27

**Authors:** Xuan Zhou, Lili Sun, Jingjing Yang, Chenlu Xia, Yuzhong Zhao

**Affiliations:** 1 Jining Polytechnic, Jining, Shandong 272007 China; 2 College of Veterinary Medicine, Shandong Agricultural University, Tai’an, Shandong 271018 China; 3 Shandong Provincial Key Laboratory of Animal Biotechnology and Disease Control and Prevention, Shandong Agricultural University, Tai’an, Shandong 271018 China

**Keywords:** Swine influenza virus, H3N2, Reassortant virus, Phylogenetic analysis, Pathogenicity

## Abstract

Swine influenza virus (SIV) not only causes significant losses to the pig industry but also poses a potential threat to human health due to its ability for cross-species transmission and zoonotic characteristics. In this study, 600 nasal swab samples were collected from pigs in Shandong Province and tested for SIV using RT-qPCR. One sample tested positive, and the virus was successfully isolated in 10-day-old specific-pathogen-free (SPF) embryonated chicken eggs. Subtype-specific RT-PCR and sequencing identified the isolate as H3N2, designated A/swine/Shandong/116/2022 (H3N2). Whole-genome sequencing and similarity analysis showed that PB2, PB1, PA, NP, and M genes were most similar to H1N1 viruses (97.71–99.67%), while HA, NA, and NS genes were closest to H3N2 viruses (96.06–97.85%), suggesting this isolate is a reassortant between H1N1 and H3N2 viruses. Phylogenetic analysis indicated that PB2, PB1, PA, NP, and M genes belong to the 2009 pandemic H1N1 (pdm/09 H1N1) lineage, HA and NA genes belong to the human-like H3N2 (HL H3N2) lineage, and the NS gene belongs to the triple-reassortant (TR) H1N2 lineage. Key amino acid analysis showed a monobasic HA cleavage site (PEKQTR/G), consistent with low pathogenicity, and residues 190V, 226I, and 228S, which may affect receptor binding. PB2 residues 271A, 590S, and 591R may influence viral replication and host adaptation. Compared with the human influenza vaccine strain A/Darwin/9/2021 (H3N2), several amino acid changes were found in HA antigenic sites A, B, C, and E, suggesting possible antigenic drift. In addition, clear differences were found in N-linked glycosylation sites between the isolate and vaccine strain, including loss of several glycosylation sites and the appearance of a new site at position 499, which may change virus antigenicity and immune recognition. Functional studies demonstrated that the isolate efficiently infected MDCK cells and replicated in the respiratory tissues of BALB/c mice, causing mild to moderate lung lesions without mortality or significant weight loss. In summary, the isolated is a multi-source reassortant virus with low pathogenicity, providing valuable insights into the genetic characteristics and epidemiology of H3N2 SIV circulating in pigs in China.

## Introduction

Swine influenza (SI) is a highly contagious respiratory disease of pigs, characterized by clinical symptoms such as fever, coughing, and respiratory distress [1]. Although most infected pigs recover rapidly, the infection is often accompanied by secondary bacterial infections [2] and may affect growth performance and productivity, causing significant economic losses in pig farming. Swine influenza virus (SIV), a member of the *Orthomyxoviridae* family, has a genome composed of eight single-stranded negative-sense RNA segments [3]. The main SIV subtypes circulating in pig populations are H1N1, H1N2, and H3N2 [4].

Notably, porcine respiratory epithelial cells express both avian-type receptors (α2,3-linked sialic acid, SA2,3Gal) and human-type receptors (α2,6-linked sialic acid, SA2,6Gal), making pigs a “mixing vessel” in which avian influenza viruses, human influenza viruses, and SIV can exchange gene segments, potentially generating new influenza viruses with pandemic potential [5].

The 2009 pandemic H1N1 virus (pdm/09 H1N1) was a novel triple-reassortant influenza A virus containing gene segments derived from human, swine, and Eurasian avian influenza viruses. The virus rapidly spread to more than 200 countries and regions, marking the first global influenza pandemic of the twenty-first century [6]. Subsequently, pdm/09 H1N1 virus was transmitted back to pigs [7] and reassorted with local H3N2 SIVs, generating H3N2 SIVs carrying internal genes from pdm/09 H1N1 [8, 9]. Multiple cases of human infection with H3N2 SIVs have since been reported [10–12]. In China, human infections with swine-origin H3N2 viruses have also been documented, for example, in 2017, researchers isolated an H3N2 SIV from a female infant in Guangdong Province [13]. These findings indicate that swine-origin H3N2 viruses continue to evolve and possess the potential for zoonotic transmission, highlighting the need for enhanced monitoring and research.

Although previous studies have reported reassortant H3N2 SIVs carrying internal genes from the pdm/09 H1N1 virus [14, 15], most of these reports are based on strains collected from southern China. In contrast, there are still limited studies on the genetic changes, mutation patterns, and biological features of recent strains from northern China. As these viruses continue to circulate in pigs, they continue to evolve and accumulate mutations. However, their current adaptation to hosts, disease characteristics, and possible ability to infect other species are still not fully clear.

In this study, we isolated and identified a reassortant H3N2 SIV from pigs in Shandong Province, China. We performed whole-genome sequencing, phylogenetic analysis, and basic biological studies, including virus growth in cells and mouse infection experiments. This study provides updated surveillance data on recent H3N2 SIVs in Shandong Province, improves understanding of their genetic diversity and evolution in China, and supports ongoing monitoring and research on SIV. These results provide descriptive evidence of the genetic and biological features of this isolate, rather than direct assessment of zoonotic risk.

## Materials and methods

### Ethics statement

All animal experiments were approved by the Animal Ethics and Experimentation Committee of Shandong Agricultural University (Shandong, China). To minimize animal suffering, all procedures were conducted by trained and qualified animal care personnel.

### Sample collection

From January to May 2022, nasal swab samples were collected from 600 pigs showing clinical signs of respiratory disease on commercial farms located in Tai’an, Jinan, Weifang, and Jining, Shandong Province, China. All sampled pig farms met the Shandong Province criteria for large-scale pig farms (i.e., > 500 pigs produced annually), with farm sizes ranging from 500 to tens of thousands of pigs per year, representing typical local production practices. The names of individual farms are not disclosed due to confidentiality agreements. Each swab was placed in 1 mL of transport medium (DMEM containing 2,000 U/mL penicillin and 2,000 U/mL streptomycin), transported to the laboratory under cold-chain conditions, and stored at − 70 °C until further use.

### Virus isolation

Frozen nasal swab samples were thawed at room temperature and vortexed thoroughly. A 200 μL aliquot of each sample was used for nucleic acid extraction. SIV was detected by real-time quantitative reverse transcription polymerase chain reaction (RT-qPCR) targeting the highly conserved matrix (M) gene of influenza A virus. Primers were designed based on conserved regions of the M gene. Amplification was performed using Taq SYBR® Green qPCR Premix (Universal) (Yugong Biotechnology, China) following the manufacturer’s instructions. Samples positive for SIV RNA were centrifuged at 2,795 × g for 5 min at 4 °C. The supernatant was inoculated into 10-day-old SPF embryonated chicken eggs (0.2 mL per egg). The inoculated eggs were incubated at 38.5 °C with 60–70% relative humidity for 72 h and then stored at 4 °C overnight. Under sterile conditions, allantoic fluid was collected from both dead and surviving embryos 24–72 h post-inoculation, aliquoted, and stored at − 70 °C. Hemagglutination titers of the allantoic fluid were determined using standard microtiter hemagglutination assays.

### Viral sequencing and genetic analysis

Viral RNA was extracted from the allantoic fluid according to the manufacturer’s instructions. Complementary DNA (cDNA) was synthesized using universal influenza A primers (12 bp: 5′-AGCRAAAGCAGG-3′). PCR amplification was performed using gene-specific primers for the eight viral segments, and the resulting amplicons were sequenced by Sangon Biotech Co., Ltd. (Shanghai, China). Sequence assembly was performed using SeqMan software, and subtype identification and nucleotide similarity analysis were conducted using the NCBI BLAST program. Amino acid variations were analyzed using MegAlign software. Potential N-linked glycosylation sites in the deduced amino acid sequence of the HA region were predicted using the NetNGlyc 1.0 Server (http://www.cbs.dtu.dk/services/NetNGlyc/). Phylogenetic analysis was performed using the neighbor-joining method implemented in MEGA version 7.0 with 1,000 bootstrap replicates. Viral sequences were compared with reference strains retrieved from the Global Initiative on Sharing Avian Influenza Data (GISAID) (https://www.gisaid.org) and the NCBI Influenza Virus Resource databases (https://www.ncbi.nlm.nih.gov/genomes/FLU/). Phylogenetic analyses were performed for each gene segment to determine their evolutionary relationships and genetic lineages.

### Determination of 50% egg infectious dose (EID_50_)

Allantoic fluid was serially diluted tenfold in DMEM containing 2,000 U/mL penicillin and 2,000 U/mL streptomycin, ranging from 10⁻^3^ to 10⁻^10^. Ten-day-old SPF embryonated chicken eggs were inoculated with 0.1 mL of each dilution, with three eggs used per dilution. Embryos were monitored every 12 h, and nonspecific deaths occurring within 24 h post-inoculation were excluded from the analysis. After 48 h, allantoic fluid was harvested and tested for hemagglutination activity. The EID_50_ value was calculated using the Reed–Muench method.

### Indirect immunofluorescence assay (IFA) and in vitro viral replication analysis

Madin-Darby canine kidney (MDCK) cells were obtained from the American Type Culture Collection (CCL‐34; ATCC, Manassas, VA, USA). MDCK cells were routinely cultured in Dulbecco’s Modified Eagle Medium (DMEM) supplemented with 10% fetal bovine serum (FBS) at 37 ℃ in a humidified incubator with 5% CO₂. Before infection, MDCK cells were seeded in 96-well plates at a density of 1–2 × 10^4^ cells per well in 100 μL medium and cultured for approximately 24 h until 90%–100% confluence was reached. The cells were then infected with A/swine/Shandong/116/2022 (H3N2) virus at a multiplicity of infection (MOI) of 0.001. After incubation at 37 ℃ for 1 h for viral adsorption, the inoculum was removed and the plates were gently rocked every 15–20 min during adsorption. After adsorption, the inoculum was discarded, and 200 μL of serum-free DMEM containing TPCK-treated trypsin (1–2 μg/mL) was added to each well. The cells were then incubated at 37 ℃ for 36 h until obvious cytopathic effect (CPE) was observed. The cells were fixed with 80% pre-cooled (− 20 ℃) acetone for 5–10 min and air-dried. Subsequently, 100 μL of a mouse monoclonal antibody against NP protein (Abcam, UK) was added to each well and incubated at 4 ℃ overnight. After washing four times with PBS, FITC-conjugated goat anti-mouse IgG (H + L) secondary antibody (SeraCare, USA) was added and incubated at 37 ℃ for 1 h in the dark. The plates were then washed again with PBS, and fluorescence signals were observed and recorded under a fluorescence microscope. To analyze viral growth kinetics, MDCK cells were infected with A/swine/Shandong/116/2022 (H3N2) virus at an MOI of 0.001 (infection procedures as described above). Culture supernatants were collected at 12, 24, 36, 48, 60, and 72 h post-infection (hpi) and stored at − 80 ℃ until use. The median tissue culture infectious dose (TCID_50_) was determined using MDCK cells, and viral titers were calculated using the Reed–Muench method. Viral growth curves were generated based on three independent experiments, and data were presented as mean ± standard deviation.

### Mouse infection experiments

Female SPF BALB/c mice (6–8 weeks old) were purchased from Speifu Biotechnology Co., Ltd. (Beijing, China). The mice were housed in a controlled animal facility with constant temperature and humidity under a 12 h light/dark cycle, with free access to food and water. To evaluate the pathogenicity and replication of H3N2 SIV in mammals, sixteen female BALB/c mice were randomly divided into an infection group and a control group (eight mice per group). Mice in the infection group were anesthetized with isoflurane (3%–5% for induction and 1%–3% for maintenance) before intranasal inoculation. When the mice became lightly anesthetized, as shown by loss of righting reflex and slow breathing, intranasal infection was performed. Each mouse received 50 µL of virus suspension containing 10^6^ EID_50_, while mice in the control group received the same volume of sterile PBS. During the procedure, anesthesia depth was carefully controlled to ensure proper delivery of the virus into the respiratory tract and to reduce stress. On day 3 post-infection (dpi), three mice were randomly selected from each group for tissue collection. Before sampling, mice were anesthetized again with isoflurane (3%–5% for induction and 1%–3% for maintenance) to ensure they were fully unconscious. Under deep anesthesia, euthanasia was performed by cervical dislocation. Death was confirmed by no breathing, no heartbeat, and no reflex response. Then lungs, nasal turbinates, spleen, kidney, and brain were collected immediately for further study. Tissues were homogenized in 1 mL of cold sterile PBS and inoculated into 10-day-old SPF embryonated chicken eggs for virus titration. Viral titers were expressed as mean ± standard deviation. The remaining mice were monitored for 14 days, and body weight and survival were recorded daily to evaluate viral pathogenicity.

### Histopathology

On 3 dpi, portions of the lung tissues were fixed in 4% paraformaldehyde for 48 h. Fixed tissues were subjected to routine dehydration, clearing, and paraffin embedding. Sections (3 µm thick) were stained with hematoxylin and eosin (H&E) and examined under a light microscope to assess histopathological changes.

## Results

### SIV detection and virus isolation

A total of 600 nasal swab samples collected from pigs were tested for SIV by RT-qPCR. Only one sample was positive for SIV. The positive sample was inoculated into the allantoic cavity of 10-day-old SPF embryonated chicken eggs for virus isolation. To purify the virus, three rounds of blind passage were performed. After serial passage, one SIV isolate was successfully obtained. The allantoic fluid from the last passage was collected and tested by a HA assay, and the HA titer was 2^7^. To check the genetic stability of the virus during isolation and passage, viral RNA was extracted from both the original positive sample and the final isolate for further genetic analysis. Subtype identification was performed using RT-PCR with primers for H1, H3, H9, N1, and N2 genes. The isolate was positive for H3 and N2, but negative for H1, H9, and N1, showing that it was H3N2. According to the standard influenza virus naming system, the isolate was named A/swine/Shandong/116/2022 (H3N2).

### Genome sequencing and sequence submission

The complete genome of the isolated H3N2 SIV was successfully sequenced, and all eight gene segments were submitted to the GenBank database. The accession numbers for each segment were as follows: PB2 (OM921396), PB1 (OM921397), PA (OM921398), HA (OM921399), NP (OM921400), NA (OM921401), M (OM921402), and NS (OM921403). Sequence comparison showed that there were no nucleotide differences between the original positive sample and the third-passage isolate in all eight gene segments, indicating that the virus was genetically stable during isolation and passage.

### Geographic and temporal distribution of H3N2 SIV isolates in China

**Fig. 1:**
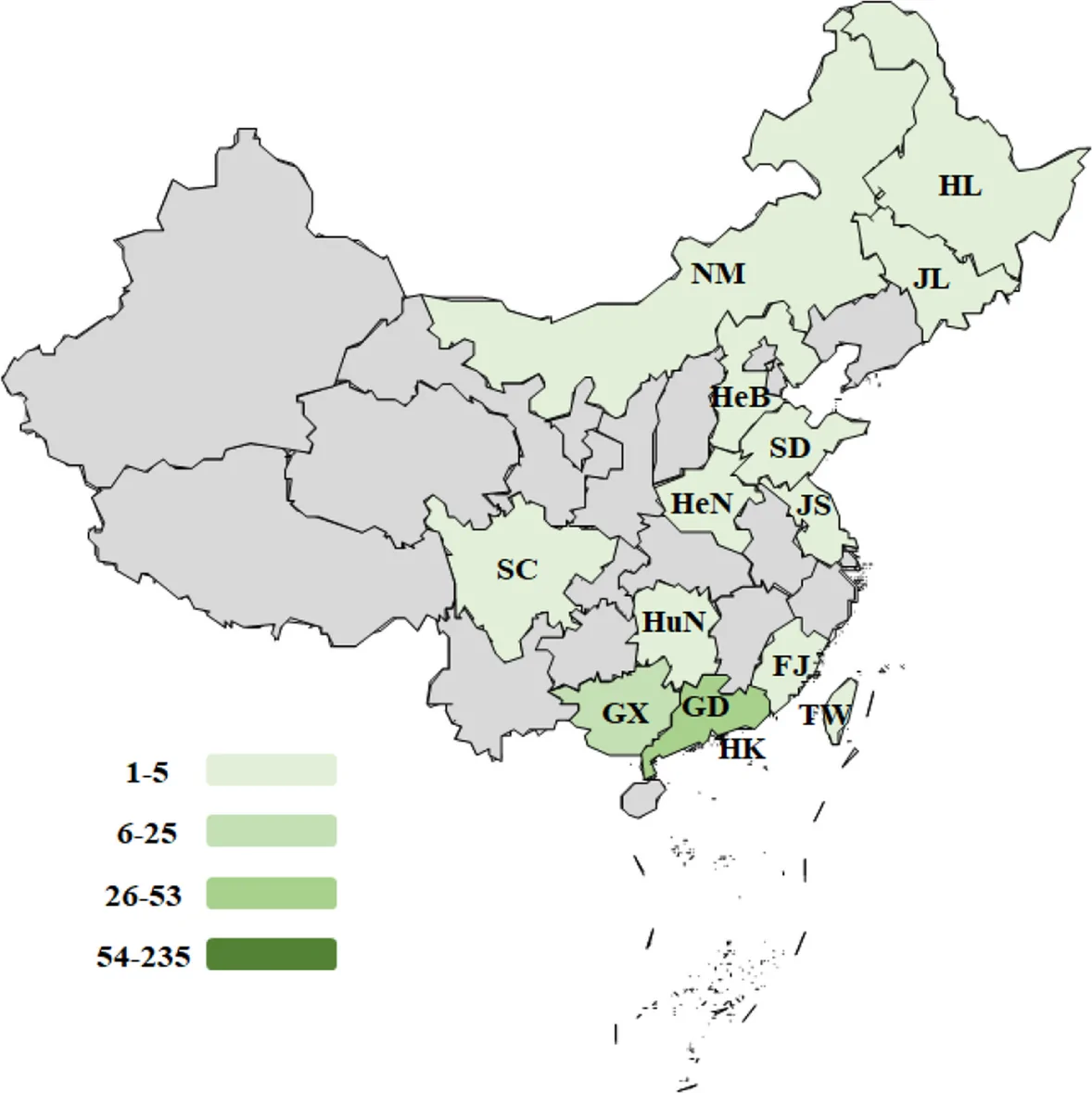
Geographic distribution of H3N2 SIVs in different regions of China. The color coding in the legend indicates different ranges of strain counts. The abbreviations correspond to the following provincial-level administrative regions: HL (Heilongjiang), JL (Jilin), NM (Inner Mongolia), HeB (Hebei), SD (Shandong), HeN (Henan), JS (Jiangsu), SC (Sichuan), HuN (Hunan), GX (Guangxi), GD (Guangdong), FJ (Fujian), HK (Hong Kong), and TW (Taiwan)

**Fig. 2:**
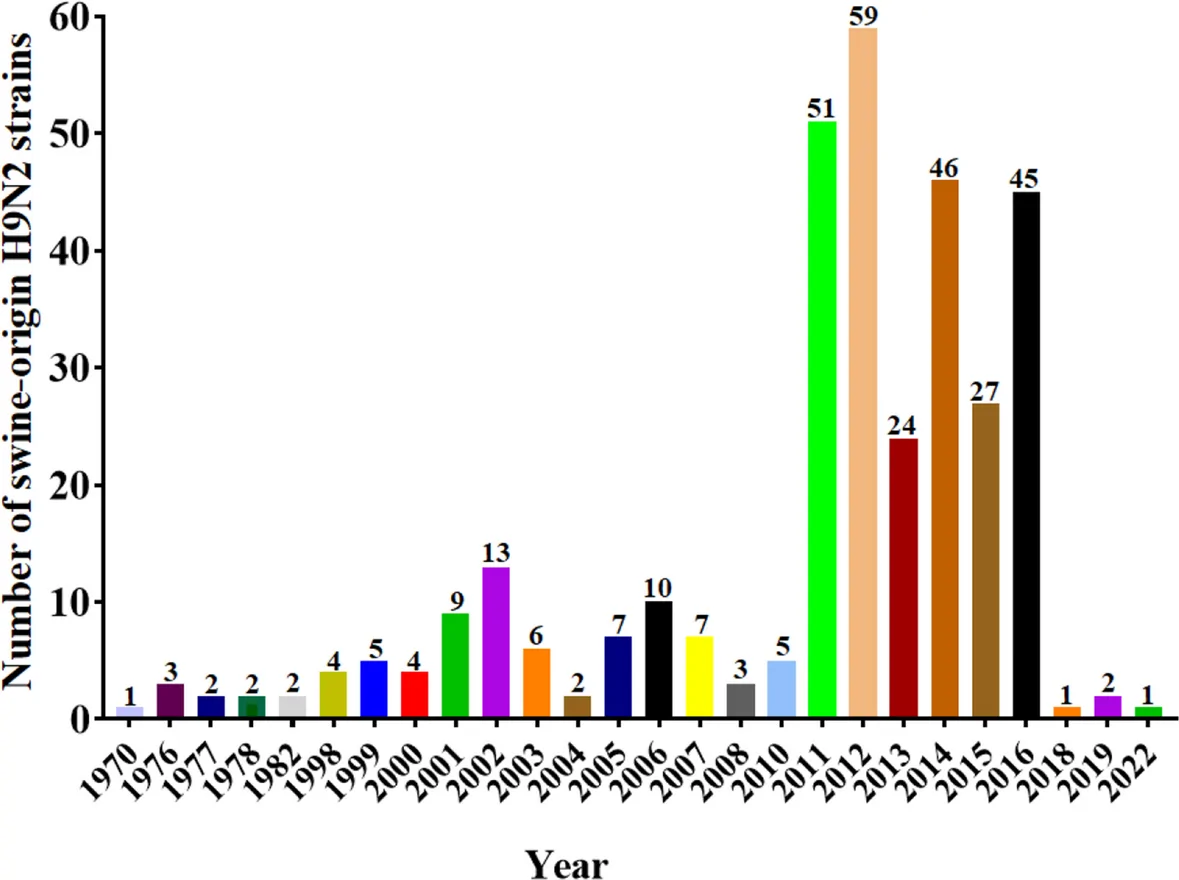
Annual distribution of H3N2 SIV strains in China, 1970–2022. The bar chart shows the number of H3N2 SIV strains recorded each year from 1970 to 2022. The number of strains peaked during 2011–2016, with the highest count (59 strains) observed in 2012

Analysis of swine H3N2 SIV isolates in China was performed using publicly available sequence data from the GISAID and the NCBI Influenza Virus Resource databases. Clear regional differences were observed in the number of isolates (Fig. 1). Hong Kong had the highest number of isolates (*n* = 235), followed by Guangdong (*n* = 53) and Guangxi (*n* = 25). All other regions had much fewer isolates (*n* ≤ 5), including Shandong, Heilongjiang, Jilin, Fujian, Taiwan, Henan, Hunan, Jiangsu, Inner Mongolia, Hebei, and Sichuan. Most isolates were mainly found in southern China, especially Hong Kong, Guangdong, and Guangxi, showing a clear geographic clustering pattern. The temporal distribution of swine H3N2 SIV isolates in China from 1970 to 2022 showed clear changes over time (Fig. 2). The number of isolates stayed low from 1970 to 2010, with slightly higher counts in 2001 (*n* = 9), 2002 (*n* = 13), and 2006 (*n* = 10). A clear increase was observed during 2011–2016, forming a major peak. The highest number was in 2012 (*n* = 59), with high numbers also in 2011 (*n* = 51) and 2016 (*n* = 45). After 2016, the number of isolates dropped quickly. Only a few isolates (≤ 2 per year) were detected from 2018 to 2022. Overall, swine H3N2 SIV in China showed long-term low-level circulation, a major increase in the early 2010 s, and a clear decline in recent years.

### Homology analysis

Genetic analysis of the eight gene segments of the isolate was performed based on nucleotide sequence homology (Table 1). The results indicated that the isolate exhibited reassortment characteristics. Overall, the internal genes showed high similarity to H1N1 SIV, whereas the surface glycoprotein genes (HA and NA) and the NS gene were more closely related to H3N2 SIV. Among the polymerase-related genes, the PB2 gene showed the closest relationship to H1N1 SIV circulating in Guangdong, Liaoning, and Shandong provinces, with a highest nucleotide identity of 98.68%. The PB1 gene was closely related to H1N1 SIV from Liaoning and Shandong, with a maximum identity of 97.85%. The PA gene showed the highest nucleotide identity of 98.65% with H1N1 viruses circulating in Liaoning. The NP gene shared high similarity with multiple human-origin H1N1 viruses, with the highest identity (99.67%) observed with A/Viet Nam/13032088/2009 (H1N1). The M gene was closely related to H1N1 SIV from Shandong and Jilin, with a maximum nucleotide identity of 98.37%. In contrast to the internal genes, the HA, NA, and NS genes were more closely related to H3N2 SIV, with highest nucleotide identities of 96.12%, 96.77%, and 97.85%, respectively. The closely related reference strains were mainly distributed in Guangxi, Guangdong, Shandong, and Hong Kong. In summary, the isolate characterized in this study is a reassortant virus derived from H1N1 and H3N2 SIV. Its genome shows a multi-origin pattern, suggesting ongoing genetic exchange among different influenza virus subtypes in swine populations.

**Table 1:** Comparison of the H3N2 SIV isolate genome with virus reference sequences in GenBank

Gene	Position	Closest Homologous Strain	Nucleotide Identity (%)
PB2	1-2280	A/swine/Guangdong/JJK/2025(H1N1)	98.68
		A/swine/Liaoning/JZ164/2020(H1N1)	98.68
		A/swine/Shandong/LY142/2017(H1N1)	98.42
PB1	1-2274	A/swine/Liaoning/FS487/2015(H1N1)	97.85
		A/swine/Shandong/SD1101/2023(H1N1)	97.80
		A/swine/Liaoning/FS176/2015(H1N1)	97.71
PA	1-2151	A/swine/Liaoning/AS1732/2020(H1N1)	98.65
		A/swine/Liaoning/HLD1706/2020(H1N1)	98.61
		A/swine/Liaoning/TL5239/2020(H1N1)	98.51
HA	1-1701	A/swine/Guangxi/2518/2011(H3N2)	96.12
		A/swine/Guangdong/1210/2012(H3N2)	96.06
		A/swine/Hong Kong/3276/2011(H3N2)	96.06
NP	1-1497	A/Viet Nam/13032088/2009(H1N1)	99.67
		A/Texas/44302533/2009(H1N1)	99.60
		A/Singapore/GP4218/2009(H1N1)	99.60
NA	1-1410	A/swine/Shandong/15/2018(H3N2)	96.77
		A/swine/Hong Kong/2789/2013(H3N2)	96.29
		A/swine/Guangxi/NS3891/2011(H3N2)	96.09
M	1-982	A/swine/Tai'an/95/2017(H1N1)	98.37
		A/swine/Jilin/21/2016(H1N1)	98.27
		A/swine/Shandong/16/2016(H1N1)	98.17
NS	1-838	A/swine/Guangxi/1874/2012(H3N2)	97.85
		A/swine/Hong Kong/362/2013(H3N2)	97.73
		A/swine/Guangxi/NS1402/2012(H3N2)	97.73
		A/swine/Hong Kong/4887/2012(H3N2)	97.73
		A/swine/Hong Kong/1331/2013(H3N2)	97.73
		A/swine/Guangxi/JGB4/2013(H3N2)	97.73

### Virus infection in MDCK cells

**Fig. 3:**
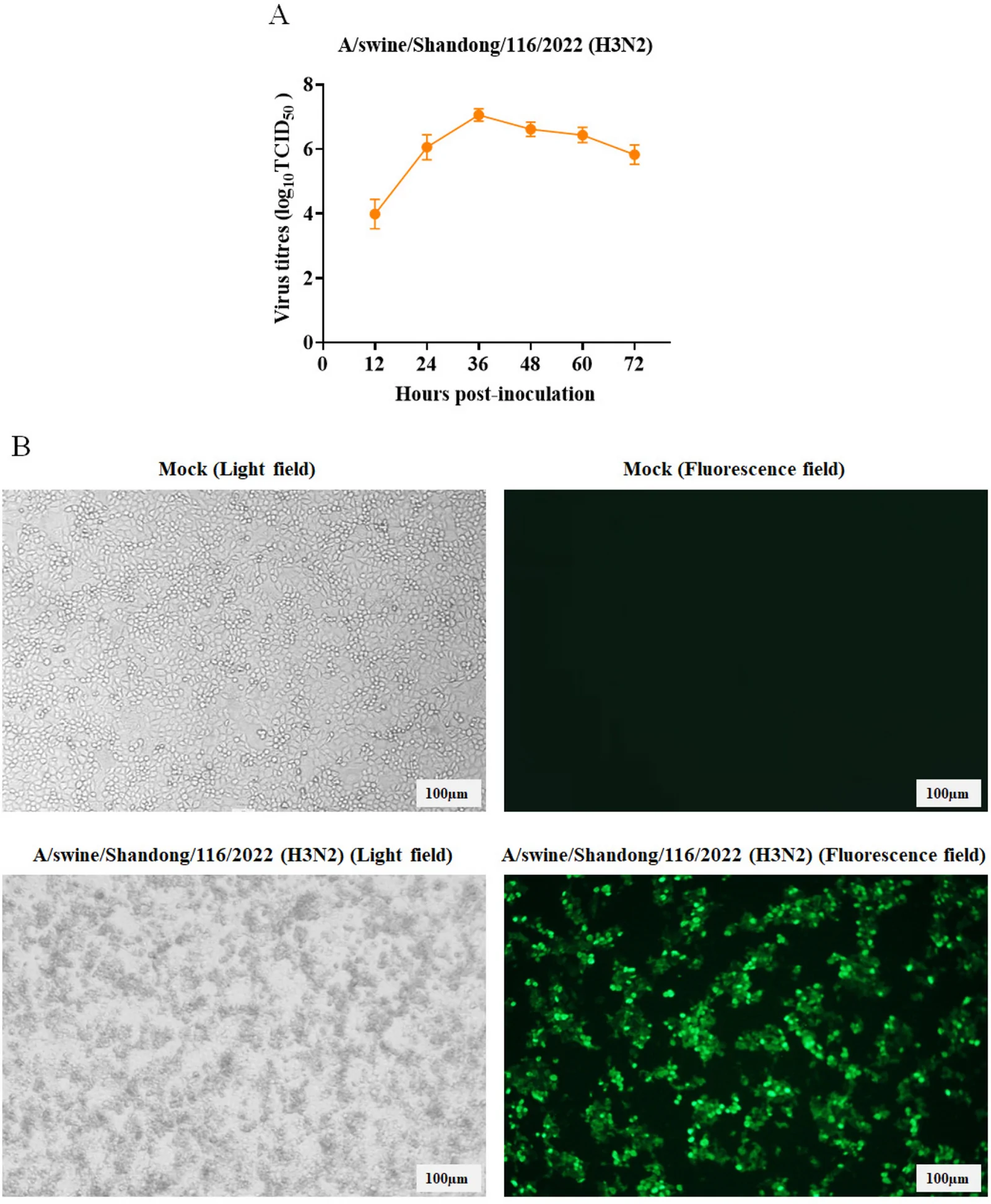
Replication characteristics of A/swine/Shandong/116/2022 (H3N2) virus in MDCK cells. **A** Growth kinetics of the virus in MDCK cells. Cells were infected at a MOI of 0.001. Supernatants were collected at 12, 24, 36, 48, 60, and 72 hpi, and viral titers were determined using the TCID_50_ assay, expressed as log_10_TCID_50_. Data are presented as the mean ± standard deviation from three independent experiments. **B** Detection of viral infection by IFA. MDCK cells were infected at a MOI of 0.001 and fixed at 36 hpi. Viral NP protein was detected using a monoclonal antibody against influenza A virus NP. Green fluorescence indicates NP-positive infected cells. The upper panel shows the mock-infected control group, and the lower panel shows the infected group. Left panels: bright-field images; right panels: fluorescence images

To evaluate the replication characteristics of A/swine/Shandong/116/2022 (H3N2) virus in vitro, MDCK cells were infected at an MOI of 0.001 and viral titers in the culture supernatants were determined by TCID_50_ assay at different time points post-infection. As shown in Fig. 3A, viral titers increased from 3.99 ± 0.45 log_10_ TCID_50_/mL at 12 hpi to a peak of 7.06 ± 0.20 log_10_ TCID_50_/mL at 36 hpi. Thereafter, viral titers gradually declined to 6.61 ± 0.23, 6.43 ± 0.24, and 5.83 ± 0.30 log_10_ TCID_50_/mL at 48, 60, and 72 hpi, respectively. At 36 hpi, obvious CPEs were observed in infected MDCK cells (Fig. 3B). IFA results showed strong influenza A virus NP protein-specific fluorescence signals in the infected group, whereas only background signals were observed in the mock-infected control group, with no detectable NP protein-specific fluorescence. These findings indicate that the virus successfully infected MDCK cells.

### Phylogenetic analysis

**Fig. 4:**
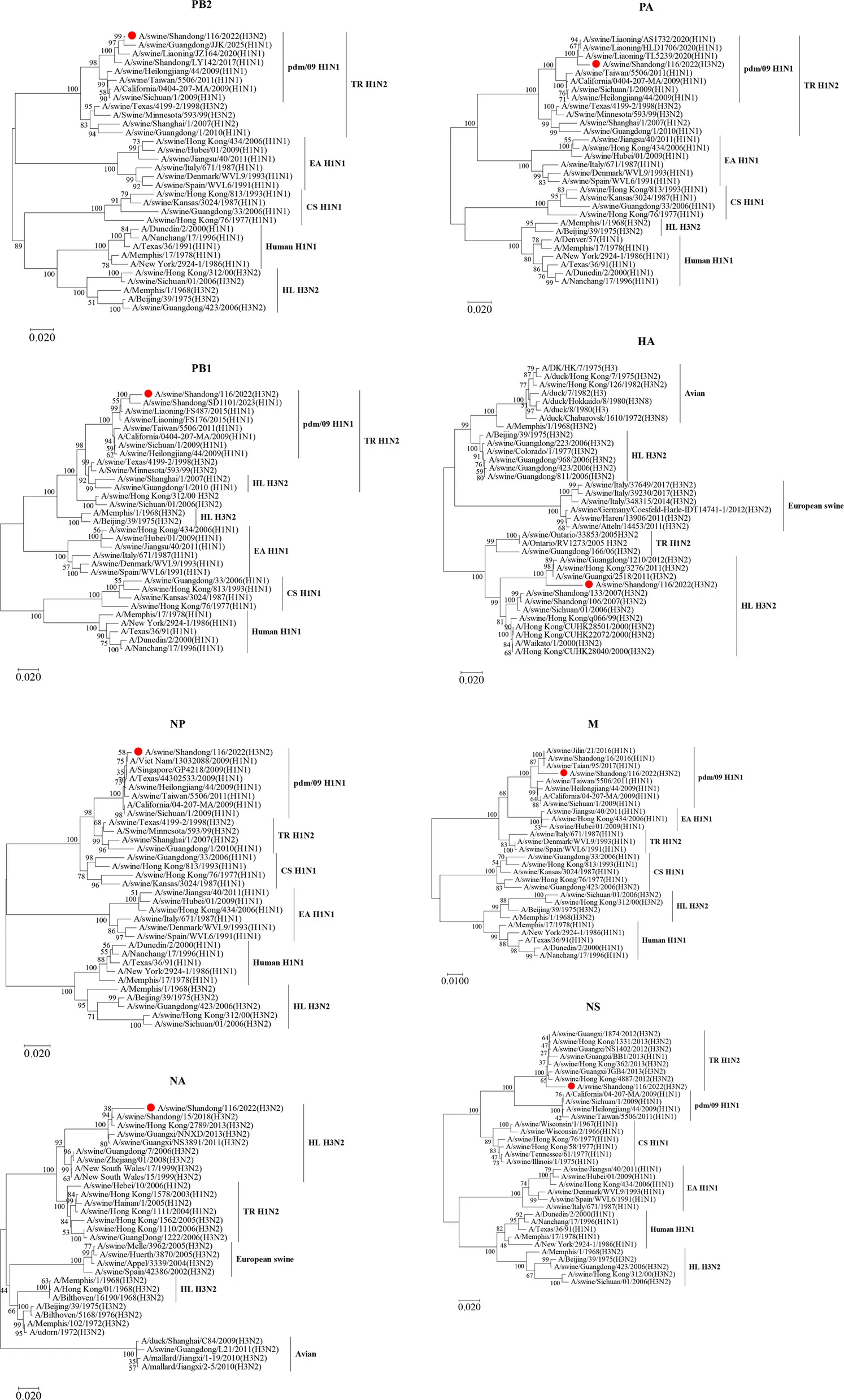
Phylogenetic analysis of the H3N2 SIV isolate. Neighbor-Joining trees based on the PB2, PB1, PA, HA, NP, NA, M, and NS genes of the isolate were constructed using MEGA 7.0 software. The isolate is shown with a red circle. Bootstrap values at branch nodes were calculated from 1000 repeats. The scale bar shows the number of nucleotide changes per site. Viral gene lineages are shown on the right side of each cluster

Phylogenetic analyses of all eight gene segments of the virus were performed according to previously described methodologies and classification criteria for influenza virus evolutionary studies [16, 17]. The results showed that phylogenetic analyses of PB2, PB1, PA, NP, M, and NS gene segments revealed that the evolutionary trees could be divided into six major lineages, including pdm/09 H1N1, classical swine H1N1 (CS H1N1), Human H1N1, Eurasian avian-like H1N1 (EA H1N1), TR H1N2, and HL H3N2. The PB2, PB1, PA, NP, and M genes of the isolate grouped with the pdm/09 H1N1 lineage, while the NS gene grouped with the TR H1N2 lineage. Phylogenetic analysis of HA and NA segments indicated that they could be classified into four lineages, including HL H3N2, avian, European swine, and TR H1N2 lineages. Both HA and NA of the isolate were assigned to the HL H3N2 lineage. Overall, the phylogenetic analysis showed that the isolate contained gene segments from different influenza virus lineages. The HA and NA genes belonged to the HL H3N2 lineage, the PB2, PB1, PA, NP, and M genes belonged to the pdm/09 H1N1 lineage, and the NS gene belonged to the TR H1N2 lineage. These results indicate that the isolate is a reassortant virus containing gene segments from the HL H3N2, pdm/09 H1N1, and TR H1N2 lineages. The detailed phylogenetic trees and branch annotations are shown in Fig. 4.

### Key amino acid analysis

To investigate potential functional mutations, the amino acid sequence of the isolated H3N2 SIV was compared with H3N2 SIV sequences retrieved from public databases at key residues associated with pathogenicity and host adaptation.

Analysis of the HA cleavage site (*n* = 335) identified six different motifs, among which PEKQTR/G was the predominant motif, accounting for 90.15% (302/335) of the sequences analyzed (Table 2). Other motifs included PDKQTR/G (4.18%), PERQTR/G (2.69%), PEKQNR/G (2.09%), PGKQTR/G (0.60%), and PEKQTK/G (0.30%) (Table 2). The cleavage site motif of the isolate was PEKQTR/G (Table 2), which is consistent with the dominant motif and characteristic of low-pathogenic avian influenza viruses [18].

**Table 2:** Key amino acid residues in the HA and PB2 proteins of the isolated virus and reference H3N2 SIVs

Protein	Position	Mutations	Isolated strain	Reference
HA	324-330 PEKQTR/G (302)	PDKQTR/G (14); PERQTR/G (9); PEKQNR/G (7); PGKQTR/G (2); PEKQTK/G (1);	PEKQTR/G	[18]
	190E (45)	D (295); G (1); N (1); V (1)	V	[19]
	226L (40)	I (284); V (15); Q (4);	I	[19]
	228S (340)	G (3)	S	[19]
PB2	271A (281)	T (2); – (23)	A	[18]
	590G (54)	S (248); N (4);	S	[18]
	591Q (55)	R (250); K (1)	R	[18]
	627E (260)	K (45); R (1)	E	[18]
	701D (298)	N (8)	D	[18]

"–" indicates no sequence at the corresponding position

Residues 190D, 226I, and 228S in the HA protein are known to influence binding to SAα−2,6-Gal [19]. Among the 343 H3N2 SIV sequences analyzed, 190D was the dominant residue (86.0%), followed by 190E (13.1%), while residues V, G, and N were each observed in only 0.3% of sequences. The isolate possessed 190V, representing a rare variation that may influence receptor-binding properties. At position 226, I was the dominant residue (82.8%), followed by L (11.7%), V (4.4%), and Q (1.2%). The isolate also possessed I at this position. At position 228, S was dominant (340/343), whereas only three sequences contained G. The isolate also carried S at this site (Table 2).

For the PB2 protein (*n* = 306), residue 271A was predominant (91.8%), while 590S (81.0%), 591R (81.7%), 627E (84.9%), and 701D (97.4%) were also the dominant residues. Previous studies have suggested that the S/R combination at positions 590/591 and the presence of 271 A may partially compensate for the absence of mammalian adaptation markers 627K and 701N [20]. The isolate contained PB2 residues 271A, 590S, 591R, 627E, and 701D (Table 2).

The HA protein of influenza A viruses can gain or lose glycosylation sites, leading to changes in viral antigenicity [22]. Prediction and comparison of potential N-linked glycosylation sites in the HA protein of the vaccine strain A/Darwin/9/2021 (H3N2) and the isolate showed clear differences in glycosylation site composition between the two strains. In the vaccine strain, residues at positions 24, 38, 54, 61, 79, 142, 149, 181, 262, and 301 were NST, NGT, NAT, NSS, NCT, NWT, NGT, NVT, NST, and NGS, respectively (Table 3). In the isolate, glycosylation motifs were found at positions 38 (NGT), 79 (NCT), 142 (NWT), 262 (NST), and 301 (NGS), and a new potential N-linked glycosylation motif (NGT) was detected at position 499 (Table 3). Comparison based on the N-X-S/T motif showed that sites 38, 79, 142, 262, and 301 were the same in both strains. In contrast, sites 24, 54, 61, 149, and 181 in the vaccine strain were not found in the isolate, indicating loss and change of glycosylation sites.

**Table 3:** Comparison of HA antigenic sites between the isolated virus and the A/Darwin/9/2021 (H3N2)

Antigenic site	Position(s)	A/Darwin/9/2021 (H3N2)	Isolated strain
A	133-137	NGTSS	NGKSA
	140-146	IRGSSSS	KRGSGNS
B	156-160	HLNYK	SLNNI
	187-198	TDKNQISLFAQS	TYDVQISLYTQA
C	51-54	EVTN	EVTN
	276-280	KCKSE	KCNSE
D	174	F	F
	207	K	K
E	63	N	N
	71	L	L
	81	N	N
	83	E	K

HA protein contains five major antigenic sites (A–E), and amino acid changes in these sites are closely related to antigenic drift [21]. Compared with the vaccine strain A/Darwin/9/2021 (H3N2), 16 amino acid substitutions were found in the HA protein of this isolate, mainly located in antigenic sites A, B, C, and E (Table 4). In antigenic site A, residues 133–137 changed from NGTSS to NGKSA, and residues 140–146 changed from IRGSSSS to KRGSGNS. In antigenic site B, residues 156–161 changed from HLNYKY to SLNNIY, and residues 187–198 changed from TDKNQISLFAQS to TYDVQISLYTQA. In antigenic site C, residues 51–54 remained unchanged, while residues 276–280 changed from KCKSE to KCNSE. In antigenic site E, residue 83 changed from E to K, while residues 63, 71, and 81 were conserved. No amino acid changes were detected at positions 174 and 207 in antigenic site D.

**Table 4:** Comparison of HA N-linked glycosylation sites between the isolated virus and A/Darwin/9/2021 (H3N2)

Position(s)	Vaccine strain-A/Darwin/9/2021 (H3N2)	Isolated strain
24	NST	/
38	NGT	NGT
54	NAT	/
61	NSS	/
79	NCT	NCT
142	NWT	NWT
149	NGT	/
181	NVT	/
262	NST	NST
301	NGS	NGS
499	/	NGT

"/" indicates no glycosylation site at the corresponding position

### Pathogenicity in mice

**Fig. 5:**
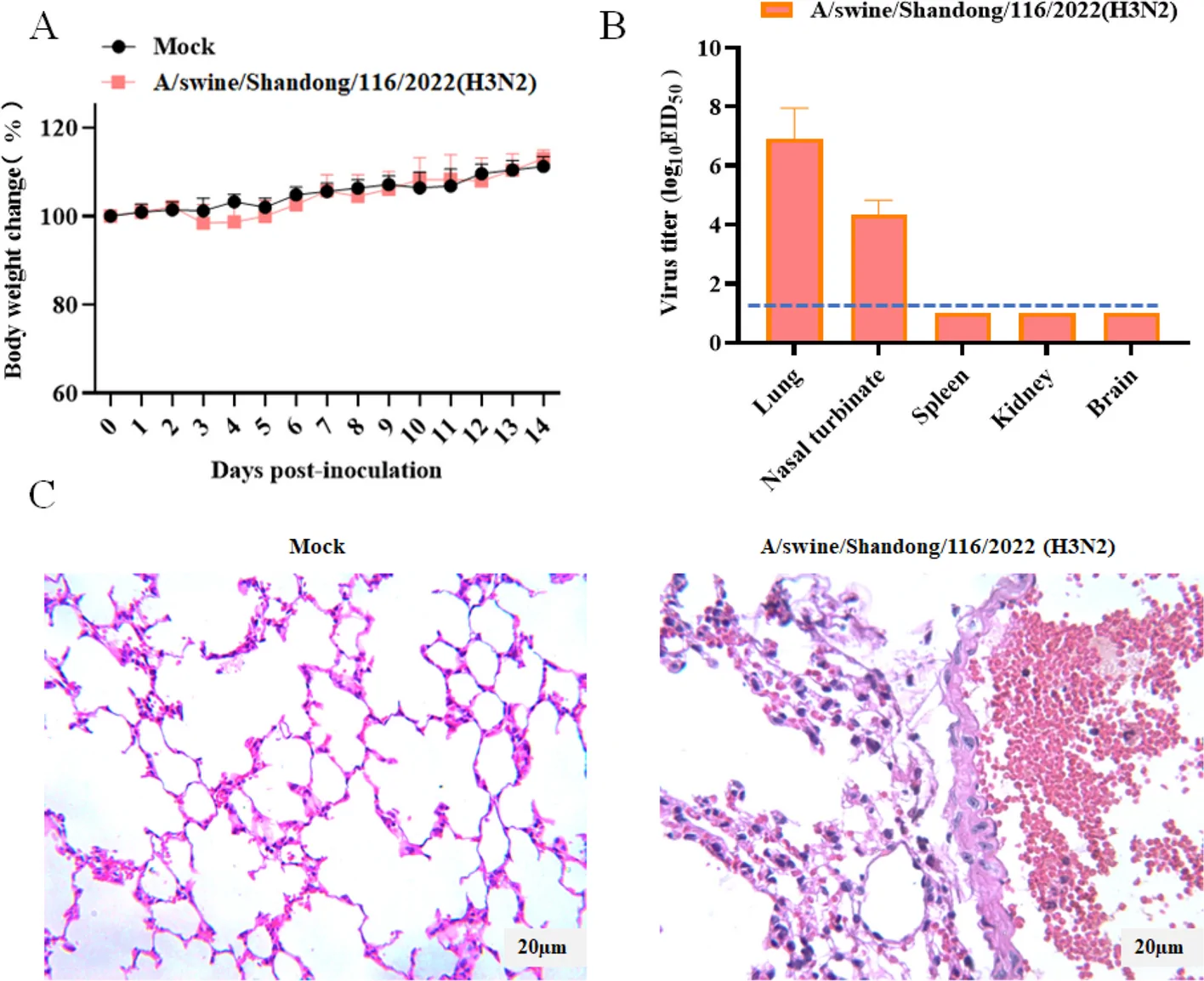
Pathogenicity of A/swine/Shandong/116/2022 (H3N2) virus in BALB/c mice. A Body weight changes in mice following intranasal inoculation monitored for 14 days. **B** Viral titers (log_10_ EID_50_/mL) in tissues collected at 3 dpi. **C** Histopathological examination of lung tissues at 3 dpi by H&E staining. Mock-inoculated mice exhibited normal lung architecture, whereas infected mice developed interstitial pneumonia with pulmonary congestion, alveolar exudation, and inflammatory cell infiltration

BALB/c mice were intranasally infected with the isolate and monitored for 14 days. Infected mice showed no significant body weight loss or mortality, with a 100% survival rate throughout the observation period (Fig. 5A), indicating limited pathogenicity in mice. At 3 dpi, viral titers were quantified in multiple tissues. The virus replicated efficiently in respiratory tissues, reaching 6.92 ± 1.04 log_10_ EID_50_/mL in the lungs and 4.36 ± 0.48 log_10_ EID_50_/mL in the nasal turbinates. In contrast, no virus was detected in the spleen, kidney or brain (Fig. 5B). Histopathological examination of lung tissues at the same time point revealed interstitial inflammatory congestion and exudative lesions, accompanied by infiltration of inflammatory cells and erythrocytes (Fig. 5C).

## Discussion

H3N2 SIV is believed to have been introduced into the Chinese swine population from human H3N2 influenza viruses as early as the 1970s–1980s [23]. Since then, the virus has spread widely among pigs and has frequently undergone genetic reassortment with other SIV subtypes during long-term co-circulation, gradually forming a dynamically evolving viral gene pool. In recent years, several studies have demonstrated that H3N2 SIV continues to circulate in Chinese swine populations and exhibits substantial genetic diversity, posing a potential threat to swine health and the pig industry [15, 24, 25].

This study performed whole-genome sequencing and phylogenetic analysis of a swine H3N2 influenza virus, A/swine/Shandong/116/2022 (H3N2), isolated in Shandong Province, China. The results showed that this virus was a reassortant virus. The PB2, PB1, PA, NP, and M genes were derived from the pdm/09 H1N1 lineage, the HA and NA genes belonged to the HL H3N2 lineage, and the NS gene originated from the TR H1N2 lineage. Together, these genes formed a virus with a pdm/09 H1N1 internal gene backbone, HL H3N2-derived HA and NA genes, and a TR H1N2-derived NS gene. Previous studies have reported similar gene patterns in different regions. A study conducted in Jiangsu Province in 2024 reported the same gene composition as that found in the present study [26]. In addition, three H3N2 SIV isolated in Guangdong in 2017 and 2018 carried HL H3N2-derived HA and NA genes together with all internal genes from the pdm/09 H1N1 lineage [15]. Similar results were also reported in Guangxi during 2013 and 2014 [17]. However, one strain contained an NS gene derived from the classical H1N1 lineage, which was different from the TR H1N2-derived NS gene identified in this study. Overall, H3N2 SIV reported in these studies commonly showed a gene pattern consisting of HL H3N2-derived HA and NA genes and a pdm/09 H1N1 internal gene backbone, while the origin of the NS gene differed among strains.

Regarding the virus isolation results, only one H3N2-positive virus was obtained from 600 respiratory samples in this study. This low detection rate should be explained in the context of the complex causes of porcine respiratory disease. Porcine respiratory disease is usually caused by more than one virus or bacteria, and it is a disease with multiple causes. In addition, influenza virus detection is affected by sampling time, sample type, and the short virus shedding period. Virus shedding usually reaches its peak within a few days after infection [27]. Therefore, virus shedding may decrease greatly when infected animals enter the subclinical stage, which reduces the chance of virus isolation. Taken together, the low isolation rate in this study may reflect both the natural features of influenza virus and the limits of field surveillance, rather than indicating a minor role of influenza virus in respiratory disease. However, because this is a surveillance-based study, other respiratory pathogens were not tested in a systematic way. Therefore, a clear cause of the observed clinical signs cannot be determined.

The ability of influenza viruses to cross species barriers is influenced by multiple factors, including the receptor-binding properties of the HA protein and the contribution of internal genes to mammalian adaptation. In this study, the amino acid combination at key HA positions (190V, 226I, and 228S) in the isolate was associated with preferential binding to SAα−2,6-Gal, suggesting a possible preference for human-type receptors [19].

In addition, the isolate contained PB2 substitutions 271A, 590S, and 591R, which have been reported to partially compensate for the absence of classical mammalian adaptation markers such as 627 K and 701 N, and may be associated with enhanced replication efficiency in mammalian hosts [20]. In 2022, the World Health Organization recommended A/Darwin/9/2021 (H3N2) as a candidate vaccine virus for the H3N2 component of seasonal influenza vaccines [18]. Antigenic analysis showed that, compared with the vaccine strain A/Darwin/9/2021 (H3N2), the HA protein of the isolate contained 16 amino acid substitutions at antigenic sites, involving multiple key antigen regions [21], indicating clear antigenic differences. These changes may reduce the effectiveness of existing vaccines against this H3N2 SIV. Further analysis showed that, compared with the vaccine strain, the virus lost several potential glycosylation sites at positions 24, 54, 61, 149, and 181, while gaining a new potential glycosylation site at position 499 [22]. These changes in glycosylation pattern may be related to reduced antibody selection pressure in the swine population.

The isolate replicated well in MDCK cells and the respiratory tract of mice, causing mild to moderate lung damage with low overall pathogenicity. These biological features are consistent with previous studies on other reassortant H3N2 viruses in mammalian models [26, 28]. Although this isolate has several molecular markers related to mammalian adaptation and can grow in mice, this study did not test its receptor-binding ability, replication capacity in human airway epithelial cells, transmission potential in ferret models, or other key factors that affect zoonotic risk. Therefore, the current results are not enough to directly evaluate its adaptability to humans or predict its possibility of spreading between humans. The molecular characteristics and replication ability observed in this study are only preliminary results that require further study, and do not serve as direct evidence of increased zoonotic risk. In addition, the mouse infection experiments were performed by intranasal inoculation of 50 μL of virus suspension. Previous studies have shown that this inoculation volume can significantly increase the proportion of particles reaching the lower respiratory tract, thereby potentially enhancing pulmonary lesions compared with natural respiratory infection, reflecting a dose-related “delivery amplification” effect [29, 30]. Therefore, the pathological findings should be interpreted with caution. Future studies using lower inoculation volumes or alternative respiratory infection models may provide a more physiologically relevant assessment of disease severity.

SIV is widely considered a potential source of future influenza pandemics because of its frequent genetic changes and reassortment events [31]. Long-term evolutionary studies of H3N2 SIV in China have shown that different viral lineages frequently exchange genes and undergo reassortment, leading to the formation of dozens of different genotypes [16]. This continuously changing reassortment network provides an important driving force for the emergence of new viral genotypes [26]. Notably, some swine-origin H3N2 viruses carry the six internal gene segments from the pdm09/H1N1 virus. These reassortant viruses are antigenically different from seasonal H3N2 viruses and show a preference for binding to human-like receptors. They can also replicate efficiently in mammalian cells, spread between guinea pigs, and infect and transmit in pigs, indicating a potential risk for cross-species transmission and zoonotic infection. The H3N2 reassortant strain isolated in this study shares a high level of overall genome similarity with previously reported high-risk reassortant viruses, with the exception of the NS gene, which comes from a different origin. In this study, we carried out genome analysis, antigen comparison, glycosylation site prediction, cell replication assays, and mouse pathogenicity experiments, which together provide a preliminary understanding of its genetic background and basic biological characteristics. However, due to experimental limitations, receptor-binding specificity tests and transmission experiments in guinea pigs were not performed. Therefore, its receptor preference and ability to transmit between mammals remain unclear. Furthermore, viral genome analysis in this study was based on Sanger sequencing, which mainly reflects consensus sequences and has limited ability to detect low-frequency variants within viral quasispecies [32]. Therefore, intrahost genetic diversity and viral quasispecies structure could not be fully analyzed. In contrast, next-generation sequencing (NGS) can detect minor variants and provides a more detailed view of viral evolution within hosts [33]. RNA virus populations are usually composed of many closely related variants within a host, forming a complex quasispecies [34]. Therefore, future studies using deep sequencing methods are needed to better describe viral genetic diversity and evolutionary changes.

Overall, this study characterized the reassortant genomic features of an H3N2 SIV circulating in pigs in Shandong Province, and evaluated its genetic characteristics, replication ability, and pathogenicity through genomic analysis and mammalian infection models. These results highlight the importance of continuous monitoring of SIV genetic diversity and reassortment events in swine populations, which helps improve our understanding of influenza virus evolution and its potential impact on animal and public health.

## Data Availability

The sequences of the strains isolated in this study have been made publicly available in the GenBank repository under accession numbers OM921396, OM921397, OM921398, OM921399, OM921400, OM921401, OM921402, and OM921403 (corresponding to the PB2, PB1, PA, HA, NP, NA, M, and NS segments, respectively).
